# Polarization-Dependent Heterodyne-Detected Sum-Frequency Generation Spectroscopy as a Tool to Explore Surface Molecular Orientation and Ångström-Scale Depth Profiling

**DOI:** 10.1021/acs.jpcb.2c02178

**Published:** 2022-07-18

**Authors:** Chun-Chieh Yu, Takakazu Seki, Kuo-Yang Chiang, Fujie Tang, Shumei Sun, Mischa Bonn, Yuki Nagata

**Affiliations:** † Max Planck Institute for Polymer Research, Ackermannweg 10, 55128 Mainz, Germany; ‡ Department of Physics, Temple University, Philadelphia, Pennsylvania 19122, United States; § Department of Physics and Applied Optics Beijing Area Major Laboratory, Beijing Normal University, Beijing 100875, China

## Abstract

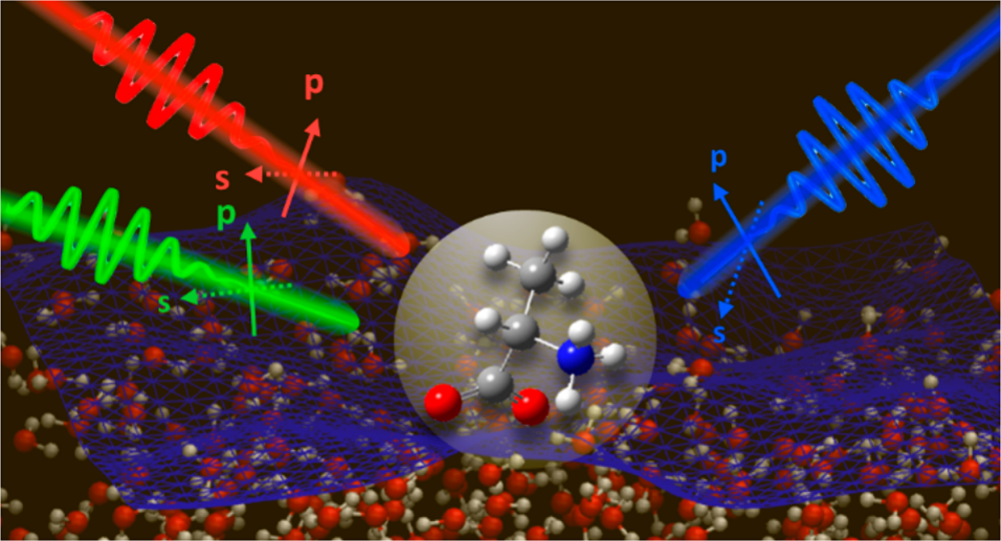

Sum-frequency generation (SFG) spectroscopy provides a unique optical probe for interfacial molecules with interface-specificity and molecular specificity. SFG measurements can be further carried out at different polarization combinations, but the target of the polarization-dependent SFG is conventionally limited to investigating the molecular orientation. Here, we explore the possibility of polarization-dependent SFG (PD-SFG) measurements with heterodyne detection (HD-PD-SFG). We stress that HD-PD-SFG enables accurate determination of the peak amplitude, a key factor of the PD-SFG data. Subsequently, we outline that HD-PD-SFG can be used not only for estimating the molecular orientation but also for investigating the interfacial dielectric profile and studying the depth profile of molecules. We further illustrate the variety of combined simulation and PD-SFG studies.

## I Introduction

The arrangement of interfacial atoms and molecules in a nanometer thickness region governs the material properties,^1^ atmospheric chemistry,^2^ chemical reactions^3^ as well as (bio)molecular processes.^4^ Understanding these processes requires knowledge of the structure of the molecules at interfaces. Among a number of the surface-specific techniques, including atomic force microscopy,^5^ X-ray spectroscopy,^6,7^ tip-enhanced Raman spectroscopy,^8^ and second-harmonic generation spectroscopy,^9^ vibrational sum-frequency generation (SFG) spectroscopy is a unique tool,^10−14^ because it allows probing the molecules at the soft matter interfaces with molecular specificity.

Vibrational SFG spectroscopy is a second-order nonlinear optical technique, and its signal is generated by the infrared (IR) and visible pulses. The SFG signal is enhanced when the IR frequency matches the frequency of vibrational mode, providing molecular specificity. The observable is the complex χ^(2)^ spectrum, where the imaginary and real parts of χ^(2)^ spectrum represent the absorption and dispersion of the molecular response, respectively. The even-order response excludes the contribution from the centrosymmetric medium, i.e., from the bulk. As such, SFG allows us to probe the interfacial molecular responses selectively, not only at solid interfaces but also at soft matter interfaces.

By carrying out SFG measurements at different polarization combinations of the IR, visible, and SFG beams, one can obtain information on the orientation of the molecular moiety of proteins, water, and organic compounds.^15−28^ Furthermore, recent studies showed that polarization-dependent (PD)-SFG has the potential to address the nature of interfacial dielectric medium^29^ and can provide depth information on the interfacial molecules.^30^ The analysis of PD-SFG requires the ratio of the peak amplitude in the Im χ^(2)^ spectra at different polarization combinations. An essential technique for accurate estimation of the Im χ^(2)^ peak amplitudes is the heterodyne detection of SFG (HD-SFG) signal,^11,31−35^ because HD-SFG can directly access the Im χ^(2)^ spectrum, unlike the conventional, homodyne-detection of SFG, which provides the |χ^(2)^|^2^ spectrum. In particular, accurate phase determination in HD-SFG^36,37^ allows us to obtain the peak amplitudes.^22,38^

In this perspective, we explain the fundamentals of the PD-HD-SFG spectra analysis and outline the research topics that the PD-HD-SFG spectroscopy can explore together with the theoretical modeling. In Section II, we introduce the principles for analyzing the PD-HD-SFG data. Section III explains the impact of the interfacial dielectric constant, which appears in the PD-HD-SFG analysis. Section IV reviews the previous orientational research using PD-HD-SFG technique. Section V describes how one can obtain the Å-scale depth information on interfacial molecules using PD-HD-SFG. In Section VI, we outline how molecular dynamics (MD) simulation can be combined with SFG spectroscopy to provide precise information on surface structure and to rationalize the interpretation of the spectra. In Section VII, we discuss the outlook for the PD-HD-SFG technique. The conclusion is given in Section VIII.

## II Polarization-Dependent Heterodyne-Detected SFG (PD-HD-SFG)

### II.A Principles of PD-HD-SFG

**Figure 1:**
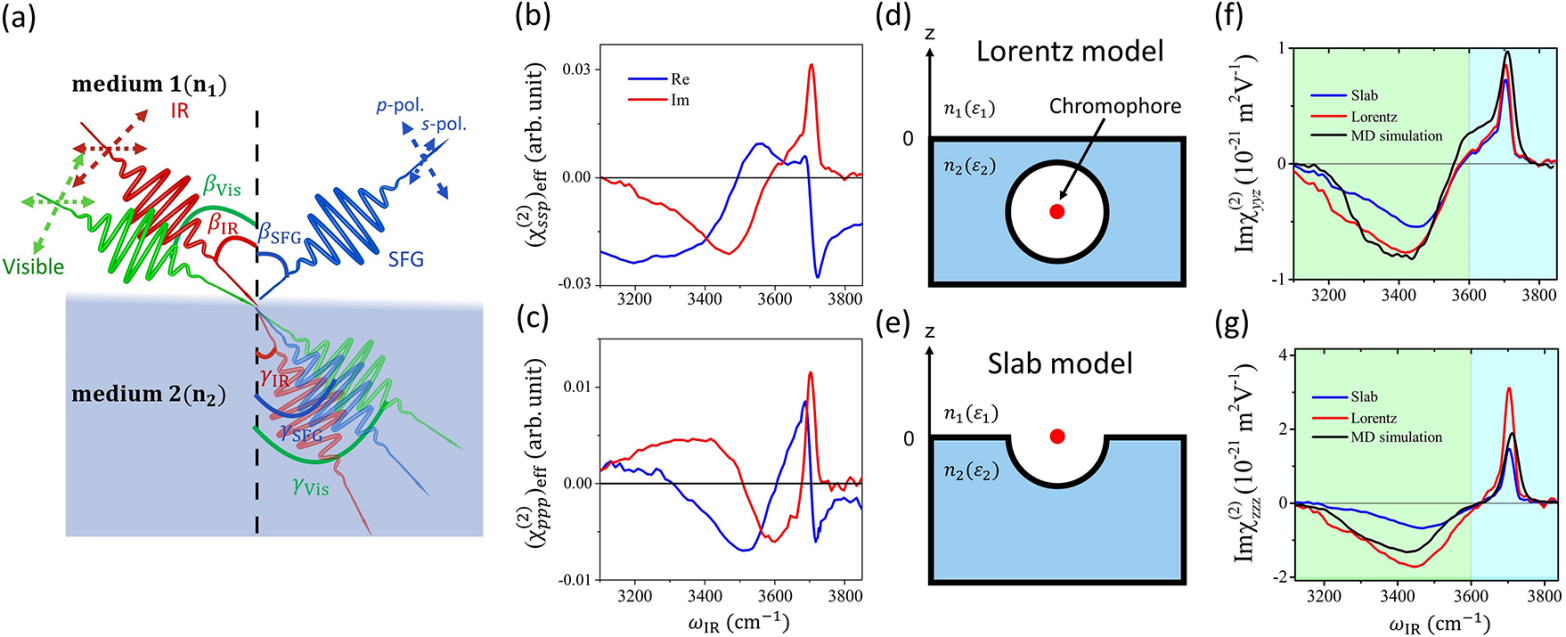
(a) Schematic of the laser beams for PD-SFG spectroscopy and their incident (β_IR_, β_Vis_), reflected (β_SFG_), and refracted (γ_IR_, γ_Vis_, γ_SFG_) angles. (b, c) Measured (χ_*ssp*_^(2)^)_eff_ (b) and (χ_*ppp*_^(2)^)_eff_ (c) spectra of the air–water interface normalized by the corresponding quartz signals. (d, e) Schematic representations of the Lorentz model (d) and the slab model (e). The vibrational chromophore (red dot) is fully solvated in part d and half-solvated in part e at the interface. (f, g) Im χ_*yyz*_^(2)^ (f) and Im χ_*zzz*_^(2)^ (g) spectra at the air–water interface obtained from the MD simulation with the POLI2VS model as well as the measured (χ_*ssp*_^(2)^)_eff_ and (χ_*ppp*_^(2)^)_eff_ spectra through the correction of the Slab model *ε′* = ε(ε + 5)/(4ε + 2), and Lorentz model *ε′* = ε. Adapted with permission from ref (29).

A number of the papers account for the measurement and processing of the HD-SFG data.^11,32,33^ Here, we explain how to take the different polarizations into account. Let us assume that we have the measured SFG spectra of the sample and the *z*-cut quartz (denoted as χ_*abc*,measured sample_^(2)^ and χ_*abc*,measure zqz_^(2)^, respectively) at the *abc* polarization for *abc* = *ssp*, *ppp*, and *sps*, where *abc* polarization represents the *a*-polarized SFG, *b*-polarized visible, and *c*-polarized IR beams. The effective SFG spectra at the *abc* polarization is given by^11^
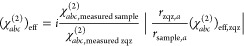
1Here, *r*_zqz,a_ and *r*_sample,*a*_ are the reflectivity coefficients of the local oscillator signal at the *z*-cut quartz and the samples surfaces for the *a*-polarized SFG beam frequency, respectively. (χ_*abc*_^(2)^)_eff,zqz_ is the effective susceptibility of the *z*-cut quartz. Assuming the crystal coordinate is overlapped with the lab coordinate, then (χ_*abc*_^(2)^)_eff,zqz_ is given by^39^
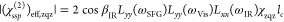
2
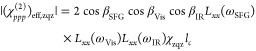
3
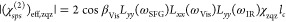
4where *β*_*i*_ and *ω*_*i*_ (*i* = IR, Vis, SFG) are the incident angle and frequency of the corresponding beam, respectively. Here, the *xz*-plane forms the incident plane of the beams, and the *z*-axis is defined as the surface normal. *l*_*c*_ is the SFG coherent length. χ_zqz_ = 8.0 × 10^–13^ m/V is the χ^(2)^ value of frequency-independent *z*-cut quartz signal.^39^*L*_*jj*_ (*j* = *x*,*y*,*z*) is the *jj* component of the Fresnel factor, and is given by
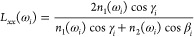
5
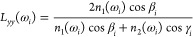
6
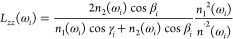
7where *γ*_*i*_ is the angle of the refracted light with its frequency of *ω*_*i*_. *n*_1_(*ω*_*i*_), *n*_2_(*ω*_*i*_), and *n′*(*ω*_*i*_) are the refractive index of bulk medium 1, bulk medium 2, and the interfacial layer, respectively. The beam configuration of the SFG measurement is displayed in Figure 1(a). Note that the dielectric constant of the interfacial layer, i.e., interfacial dielectric constant, *ε’* (*ω*_*i*_) = *n′*^2^ (*ω*_*i*_) is not known and thus should be obtained from the model calculation^16^ or simulation.^40^ Note that *L*_*zz*_ does not appear for the χ^(2)^ signal for the *z*-cut quartz in eqs 2-4, while it plays a critical role when we obtain the χ^(2)^ signal for the sample.

### II.B Analysis of PD-HD-SFG

The *yyz*-, *zzz*-, and *yzy*-components of the χ^(2)^ spectra are obtained from the measured (χ_*ssp*_^(2)^)_eff_, (χ_*ppp*_^(2)^)_eff_, and (χ_*sps*_^(2)^)_eff_ spectra via
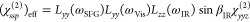
8
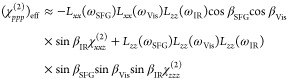
9
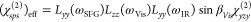
10

Note that eq 9 is valid when the χ_*xzx*_^(2)^ and χ_*zxx*_^(2)^ components are negligibly small compared to the χ_*xxz*_^(2)^ and χ_*zzz*_^(2)^ components. For a case where *x* and *y* axes are indistinguishable (as for liquid interfaces), we have χ_*xxz*_^(2)^ = χ_*yyz*_^(2)^. Under the situation that the vibrational relaxation is slower than the rotational motion of vibrational chromophores (slow-motion limit),^41^ the peak amplitudes of the vibrational modes with the *C*_∞*v*_ symmetry group (such as the free O–H group of water, and C–H and C=O stretches of formic acid) in the χ_*yyz*_^(2)^, χ_*zzz*_^(2)^, and χ_*yzy*_^(2)^ spectra (denoted as *A*_*yyz*_^(2)^, *A*_*zzz*_^(2)^, and *A*_*yzy*_^(2)^, respectively) are connected via the following forms:
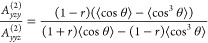
11
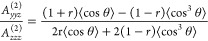
12where *r* denotes the depolarization ratio for the target vibration, and θ represents the angle formed by the molecular axis and surface normal. Equations 11 and 12 are the key equations for orientational analysis and depth analysis.

The depolarization ratio of *r* has been obtained either from the Raman data^41−43^ or from *ab initio* calculations.^44−46^ The recent development of *ab initio* calculation allows us to obtain the *r* value directly.

## III Impact of Interfacial Dielectric Constant

Here, we examine the impact of the interfacial dielectric constant by using the SFG spectra at the air–water interface based on our recent paper.^29^ The (χ_*ssp*_^(2)^)_eff_ and (χ_*ppp*_^(2)^)_eff_ spectra are displayed in parts b and c of Figure 1, respectively. The signs of the responses contained in the Im χ^(2)^ spectra reflect the absolute orientation of molecular moieties; for the O–H stretch, for example, the positive (negative) sign of the peak indicates that the O–H group points *up* to the air (*down* to the bulk water).^47,48^ Specifically, the Im(χ_*ssp*_^(2)^)_eff_ spectrum shows the positive ∼3700 cm^–1^ peak and the negative band ranging from 3000 to 3500 cm^–1^, indicating that the dangling O–H group points toward the air, while the hydrogen-bonded O–H group points toward the bulk.^49−53^

To convert the (χ_*ssp*_^(2)^)_eff_ and (χ_*ppp*_^(2)^)_eff_ spectra into the (χ_*yyz*_^(2)^)_eff_ and (χ_*zzz*_^(2)^)_eff_ spectra, two approaches have been commonly adopted to describe the interfacial dielectric constant *ε′*: fully embedded model (Lorentz model) and half-embedded model (Slab model).^16^ In the Lorentz model, where the vibrational chromophores are fully solvated (Figure 1d), *ε′* = ε. In the half-embedded model, the vibrational chromophores are half-solvated (Figure 1e), and the interfacial dielectric constant can be expressed by . Parts f and g of Figure 1 display Im (χ_*yyz*_^(2)^)_eff_ and Im (χ_*zzz*_^(2)^)_eff_ spectra with the fully- and half-embedded model of *ε′*. The comparison between the simulated and experimental Im χ^(2)^ spectra indicates that neither the Lorentz model nor the Slab model provides satisfactory agreement. The Lorentz model overestimates the 3700 cm^–1^ peak amplitude in the Im (χ_*zzz*_^(2)^)_eff_ spectrum, while the Slab model underestimates the amplitude of the negative 3100–3500 cm^–1^ band in both Im (χ_*yyz*_^(2)^)_eff_ and Im (χ_*zzz*_^(2)^)_eff_ spectra. Such disagreement between the experimental data and simulation data indicates that the choice of models has a strong impact on the concluded molecular response inferred from experimental Im χ^(2)^ spectra.

Next, we examine the impact of the model of the interfacial dielectric constant (*ε′*) on the values of *A*_*yzy*_^(2)^/*A*_*yyz*_^(2)^ and *A*_*zzz*_^(2)^/*A*_*yyz*_^(2)^ in the C–H stretch mode (∼2900 cm^–1^) region by assuming that a single formic acid molecule is located at the air–water interface, and thus, the dielectric profile is governed by water.^22^ Here, we set the incident angles of 64° and 50° for visible and IR beams, respectively, and the visible wavelength of 800 nm, eqs 11 and 12 can be recast as
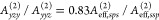
13
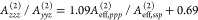
14in the half-embedded model, while they are given by
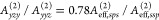
15
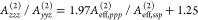
16in the fully embedded model. The comparison of eqs 13 and 14 and eqs 15 and 16 clearly shows that *A*_*yzy*_^(2)^/*A*_*yyz*_^(2)^ is much less sensitive to the choice of model to describe the interfacial dielectric constant, than *A*_*zzz*_^(2)^/*A*_*yyz*_^(2)^.

## IV Orientational Analysis and (Multidimensional) Orientation Distribution

### IV.A Principles of Orientational Analysis

As is seen in eqs 11 and 12, one can obtain ⟨cos θ⟩/⟨cos^3^ θ⟩ from either *A*_*yzy*_^(2)^/*A*_*yyz*_^(2)^ or *A*_*yyz*_^(2)^/*A*_*zzz*_^(2)^, while it is highly recommended to use *A*_*yzy*_^(2)^/*A*_*yyz*_^(2)^ rather than *A*_*yyz*_^(2)^/*A*_*zzz*_^(2)^ for obtaining ⟨cos θ⟩/⟨cos^3^ θ⟩, because *A*_*yzy*_^(2)^/*A*_*yyz*_^(2)^ is practically insensitive to the interfacial dielectric constant, as is discussed in Section III. Since the quantity ⟨cos θ⟩/⟨cos^3^ θ⟩ does not directly provide the physical insights into the orientation, one may want to convert ⟨cos θ⟩/⟨cos^3^ θ⟩ to ⟨θ⟩. To do so, one needs to assume the orientational distribution function *f*(θ_1_,···,*θ*_*n*_). The ensemble average of B is given by:
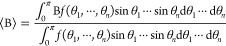
17Below, we consider a one-dimensional orientational distribution function *f*(θ) for simplicity. So far, four distinct orientation distribution functions have been assumed: the rectangular function (eq 18),^54−56^ the Gaussian-shaped function (eq 19),^57−59^ the delta function (eq 20),^15,19,60−62^ and the exponential decay function (eq 21):^45^
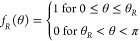
18
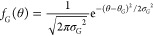
19
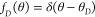
20
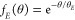
21

**Figure 2:**
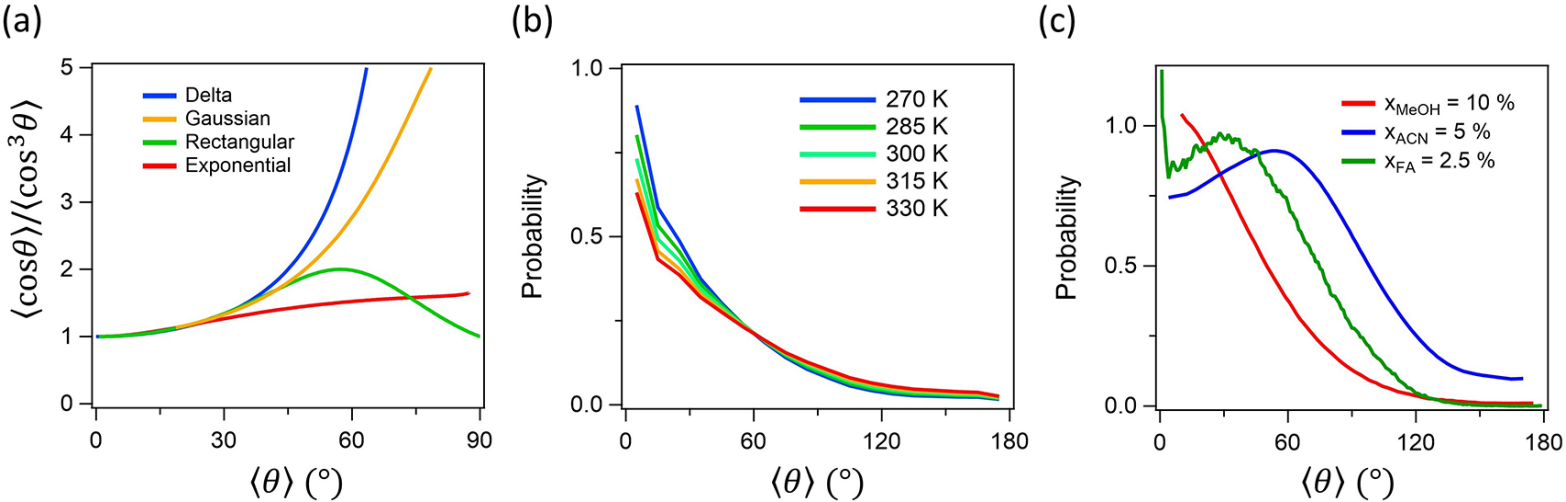
(a) ⟨cos θ⟩/⟨cos^3^ θ ⟩ vs. ⟨θ⟩ based on different distribution functions *f*_*D*_(θ), *f*_*R*_(θ), *f*_*E*_(θ), and *f*_*G*_(θ). *f*_*G*_(θ) employs *σ*_*G*_ = 15°.^57^ (b) Orientational distribution of the free O–H group of water with respect to the surface normal at the air–water interface obtained from the classical MD simulation. Adapted with permission from ref (45). Copyright 2018 American Physical Society. (c) Orientational distributions of C–H stretch of formic acid^22^ and C–H symmetric stretch of methanol^64^ and acetonitrile.^46^ Definition of the angle θ formed by the molecular axis and the surface normal. Adapted with permission from refs (22) (Copyright 2022 AIP Publishing), (46) (Copyright 2017 Royal Society of Chemistry), and (64) (Copyright 2015 American Chemical Society), respectively.

Because the choice of the orientational distribution function critically affects the inferred molecular orientation,^45,46,52^ as illustrated in Figure 2a, it is important to check the shape of *f*(θ) with MD simulations (with multiple force field models in the classical MD simulation^45^ or accurate MD techniques including *ab initio* MD (AIMD)^22,63^). In fact, the shapes of *f*(θ) computed by MD simulation indicates that it is challenging to predict the functional form of *f*(θ).

Although these functions are approximations, and none of the functions can be used universally to obtain the interfacial molecules’ angular information, we have the following pointers based on previous studies.

1. Rectangular (*f*_*R*_(θ)) and exponential decay (*f*_*E*_(θ)) functions are the approximations on the basis of the broadness of the distribution, while the Gaussian ((*f*_*G*_(θ)) and delta ((*f*_*D*_(θ)) functions focus on the center angle.
2. *f*_*G*_(θ) and *f*_*D*_(θ) seem more appropriate for some large molecules at the liquid interfaces^21,26^ or self-assembled molecules on solid surfaces,^59^ while the use of *f*_*R*_(θ) and *f*_*E*_(θ) appears more suitable for modeling the angle distribution of a small molecule.^22,45^

### IV.B Free O–D Group of Water at Air–D_2_O Interface

**Figure 3:**
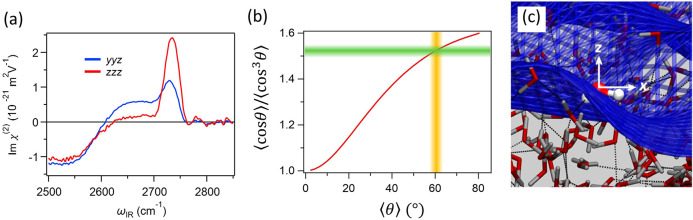
(a) Im χ_*yyz*_^(2)^ and Im χ_*zzz*_^(2)^ spectra at the air–D_2_O interface. The Slab model *ε′* = ε(ε + 5)/(4ε + 2) was used for the Fresnel factor correction (eqs 8 and 9). (b) ⟨cos θ⟩/⟨cos^3^θ⟩ vs. ⟨θ⟩ based on the exponentially decayed distribution function *f*_*E*_(θ) together with experimentally determined ⟨cos θ⟩/⟨cos^3^θ⟩ and ⟨θ⟩. (c) Snapshot of the simulated air–water interface representing the interfacial water structure on the capillary wave. Reprinted with permission from ref (45). Copyright 2018 American Physical Society.

Here, we explain how an orientational analysis can be done using PD-HD-SFG, by revisiting the orientation of the free O–D group at the air-D_2_O interface.^45^ Note that we used *A*_*yyz*_^(2)^/*A*_*zzz*_^(2)^ rather than *A*_*yzy*_^(2)^/*A*_*yyz*_^(2)^, because the χ_*yzy*_^(2)^ contribution is extremely small.^41,45,57^Figure 3a shows the Im χ_*yyz*_^(2)^ and Im χ_*zzz*_^(2)^ spectra at the air-D_2_O interface. Both spectra commonly show the dangling O–D stretch mode at ∼2730 cm^–1^ and antisymmetric mode of water molecules with one weak donor bonded O–D at ∼2650 cm^–1^. To extract the free O–D contributions in the Im χ_*yyz*_^(2)^ and Im χ_*zzz*_^(2)^ spectra (denoted as *A*_*yyz*_^(2)^ and *A*_*zzz*_^(2)^, respectively), we fitted Gaussian lineshapes to the spectra. These fits provide the amplitude ratio of *A*_*yyz*_^(2)^/*A*_*zzz*_^(2)^ of ∼0.42 for the dangling O–D stretch mode. Through eq 12 with *r* = 0.15,^45^ we obtained ⟨cos θ⟩/⟨cos^3^ θ⟩ ≅ 1.52. When we use *f*_*E*_(θ) for the orientational distribution (eq 21), which resembles the distributions obtained from the MD simulation (see Figure 2b),^45^ ⟨cos θ⟩/⟨cos^3^ θ⟩ ≅ 1.52 provides ⟨θ⟩ ≅ 59°, as is seen in Figure 3b.

The broad exponential distribution function for the free O–D group shows that ∼20% of the free O–D groups at the air–D_2_O interface point down to the bulk. The presence of the free O–D groups pointing *down* to the bulk can be ascribed to capillary waves causing surface roughness. While on the top and bottom of the capillary wave, a free O–D group typically points *up*, on the slope of the capillary wave, the free O–D groups have the tendency to point down. A snapshot of an MD simulation clearly captures this behavior of the free O–D group (Figure 3c). As such, due to the surface nanoroughness, the distribution of the free O–D groups becomes much broader and exponential shape.

### IV.C Formic Acid Molecule at the Air–Water Interface

As is seen in Figure 2c, the molecular distribution of acetonitrile and formic acid molecules at the air–water interface cannot be described by any of the functions given in eqs 18–21. How should we extract the molecular orientation from the PD-HD-SFG data? The complicated distribution function often arises from the competing driving forces to stabilize the molecular structure at interfaces. For formic acid, the two oxygen atoms and one hydrogen atom generate the competing driving forces, i.e., multiple types of hydrogen bonds with water molecules. In such a case, a multidimensional orientational distribution function (or joint-probability function)^21,22^ should be considered, rather than an orientational distribution function as a function of a single orientation parameter. One can determine the multidimensional distribution function via the multimode SFG probe.^65,66^ Below, we outline how to extract the molecular orientation using the multidimensional orientational distribution functions by focusing on formic acid molecules at the air–water interface.

We assume that the multidimensional orientational distribution function for the formic acid molecule can be given as
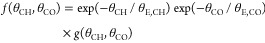
22by assuming an exponential decay function, where θ_E_ is a parameter determining the steepness/width of the exponential decay function and *g*(θ_CH_, θ_CO_) represents the geometric constraint which θ_CH_ and θ_CO_ should satisfy. The term *g*(θ_CH_, θ_CO_) is needed because the orientations of the C–H group and C=O group are not independent for a formic acid molecule; the intramolecular H–C=O angle is ∼120°.

**Figure 4:**
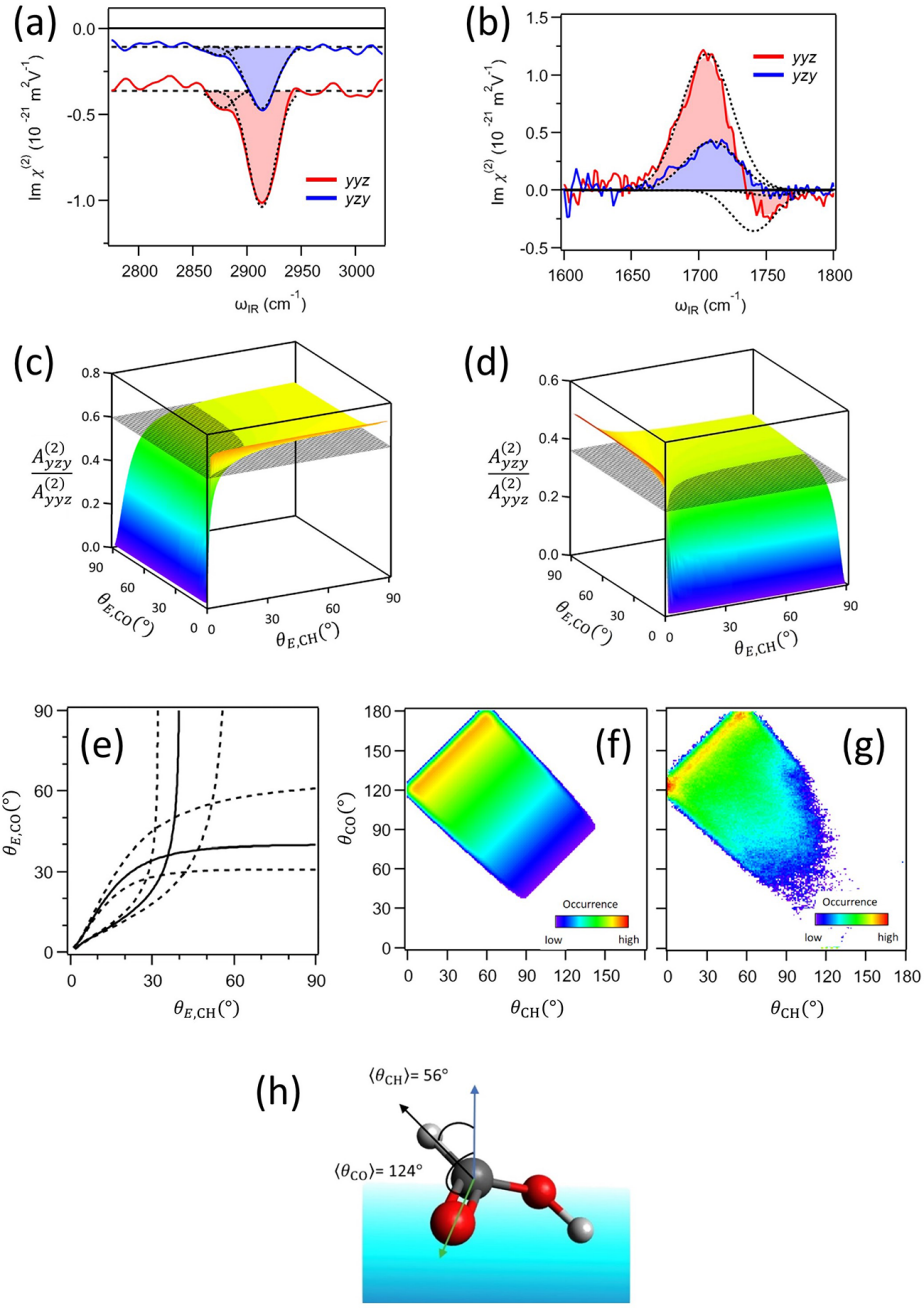
(a, b) Im χ_*yyz*_^(2)^ and Im χ_*yzy*_^(2)^ spectra in the C–H stretch mode (a) and C=O stretch mode (b) regions. The dotted lines represent the Gaussian lineshapes obtained from the fit, while the filled area represents the sum of the two Gaussians. (c, d) *A*_*yzy*_^(2)^/*A*_*yyz*_^(2)^ vs θ_E,CH_ and θ_E,CO_ for the C–H stretch mode (c) and the C=O stretch mode (d). The rainbow 3D curves represent the numerical data based on eq 11, while the gray planes represent the experimental values. (e) Lines obtained from the crossing of rainbow 3D curves and gray planes in parts c and d. The dotted lines represent the experimental error. (f) The 2D orientational distributions inferred from the crossing point of part e. (g) 2D orientational distribution obtained from the AIMD simulation. (h) Schematic of the average orientation of a formic acid molecule at the air–water interface. The blue arrow represents the surface normal. The black and green arrows represent the C → H and C → O vectors, respectively. Reprinted with permission from ref (22). Copyright 2022 AIP Publishing.

The Im χ_*yyz*_^(2)^ and Im χ_*yzy*_^(2)^ spectra of the C–H and C=O stretch modes of interfacial formic acid are displayed in parts a and b of Figure 4, respectively. From these spectra, we determined the ratio of *A*_*yzy*_^(2)^/*A*_*yyz*_^(2)^ to be 0.60 ± 0.01 for the C–H stretch mode and 0.36 ± 0.01 for the C=O stretch mode. By using eqs 11, 17, and 22, we can determine the parameter of θ_E,CH_ and θ_E,CO_. Parts c and d of Figure 4 display the *A*_*yzy*_^(2)^/*A*_*yyz*_^(2)^ values calculated for various θ_E,CH_ and θ_E,CO_ via eqs 11, 17, and 22 (rainbow curves), as well as the experimentally determined *A*_*yzy*_^(2)^/*A*_*yyz*_^(2)^ (gray planes). The crossing lines of the rainbow curves and gray planes in parts c and d of Figure 4 represent the conditions that θ_E,CH_ and θ_E,CO_ should satisfy in the C–H and C=O stretch modes, respectively. By coupling these crossing curves, one can find a crossing point (Figure 4e).

The orientational distribution obtained from the above-mentioned procedure is displayed in Figure 4f, and shows good agreement with that obtained from the AIMD simulation data (Figure 4g). This good agreement demonstrates that the multimode coupling scheme can accurately predict the orientation of the formic acid molecules. The obtained distribution functions provide ⟨θ_CH_⟩ = 56 ± 5° and ⟨θ_CO_⟩ = 124 ± 5°. The summary of the *trans*-conformation of the interfacial formic acid molecule is shown in Figure 4h. The multimode PD-HD-SFG technique using the multidimensional orientational distribution provides a universal approach for obtaining the interfacial molecular orientation. This method can also be applied to the biomolecules by probing the different moieties of the amino acid unit.

## V Å-Scale Depth Information Mediated by Interfacial Dielectric Constant

Above, we learned that we can obtain the χ_*yyz*_^(2)^, χ_*zzz*_^(2)^, and *x*_*yzy*_^(2)^ spectra from the measured (χ_*ssp*_^(2)^)_eff_, (χ_*ppp*_^(2)^)_eff_, and (χ_*sps*_^(2)^)_eff_ spectra via eqs 8–10. On the other hand, the peak amplitudes in the Im χ_*yyz*_^(2)^, Im χ_*zzz*_^(2)^, and Im χ_*yzy*_^(2)^ spectra of *A*_*yyz*_^(2)^, *A*_*zzz*_^(2)^, and *A*_*yzy*_^(2)^, respectively, are not independent; *A*_*yyz*_^(2)^, *A*_*zzz*_^(2)^, and *A*_*yzy*_^(2)^ are related via eqs 11 and 12. Now, let us focus on the *A*_*zzz*_^(2)^ value. The value for *A*_*zzz*_^(2)^ can be obtained using two different routes; one route is to acquire the *A*_*yyz*_^(2)^ and *A*_*yzy*_^(2)^ values from the Im χ_*yyz*_^(2)^ and Im χ_*yzy*_^(2)^ spectra and sequentially obtain the *A*_*zzz*_^(2)^ value based on eqs 11 and 12. The other route is to obtain the *A*_*zzz*_^(2)^ value directly from the Im χ_*zzz*_^(2)^ spectra. The values of *A*_*zzz*_^(2)^ obtained from these routes are not necessarily identical, because they depend on the choice of interfacial dielectric constant (ε′). Inversely, through the comparison of *A*_*zzz*_^(2)^, one has access to the interfacial dielectric constant. As such, one can determine ε′ through the matching of two *A*_*zzz*_^(2)^ values. Note that the same analysis can also be done with a focus on the *A*_*yyz*_^(2)^ and *A*_*yzy*_^(2)^ values. Next, we explain how to explore the depth information from our recent work.^30^

**Figure 5:**
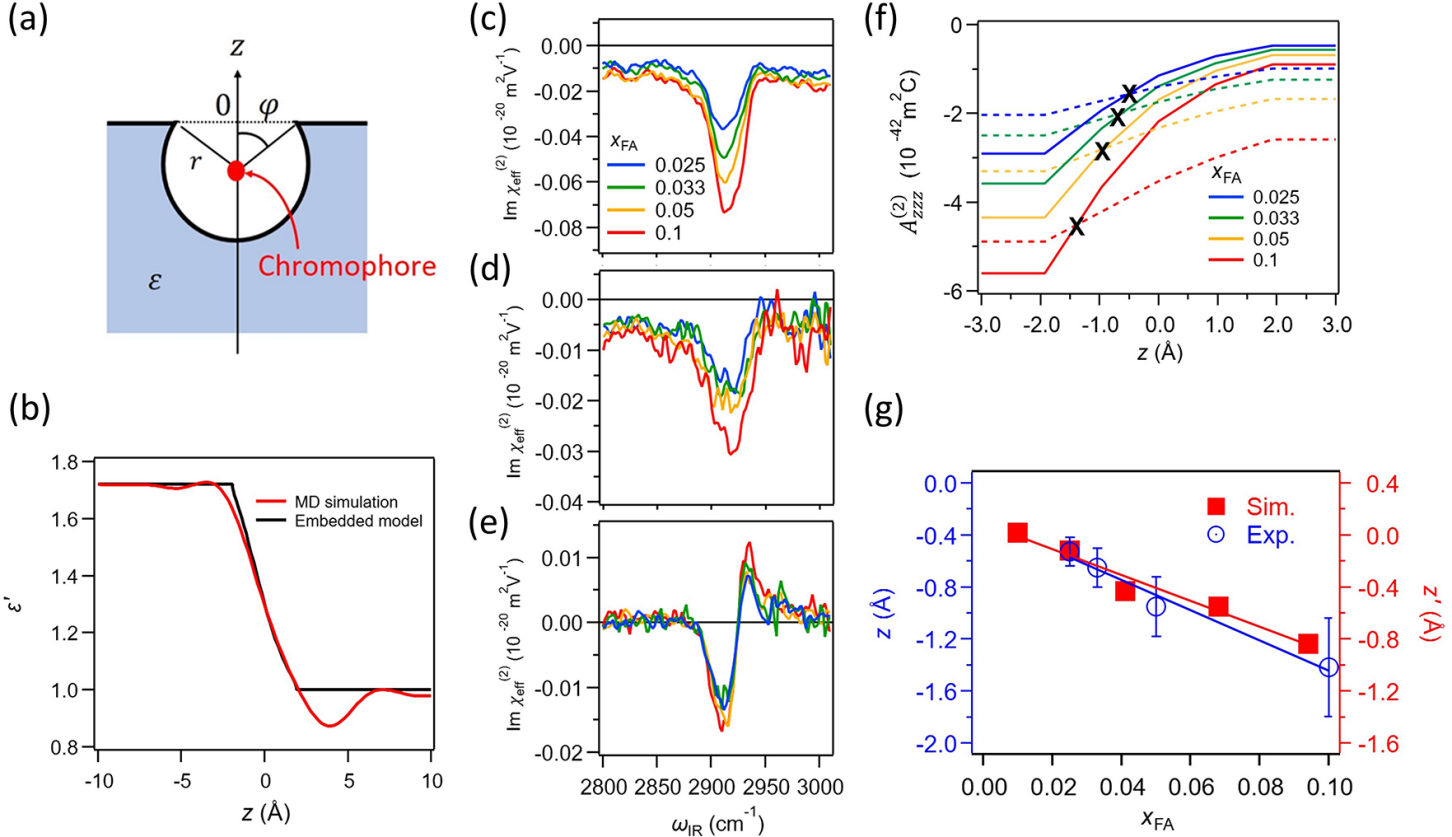
(a) Schematic representation of a vibrational chromophore (red dot) at the interface. *r* is the radius of the vibrational chromophore, and φ is the angle between the surface normal and the vector pointing from the center of the sphere to the crossing point of the dielectric interface and the surface of the sphere. The origin point of the *z*-axis is the position where the chromophore experiences *ε’* = ε(ε + 5)/(4ε + 2).^16^ (b) Depth profile of *ε′* at the air–water interface in the optical limit (ε = 1.72). The black line is obtained from eq 23, while the red line represents the MD simulation result reproduced from ref (40). Note that the impact of the surface roughness was removed from the density profile of *ε′* through the deconvolution. (c–e) Measured Im (χ_*ssp*_^(2)^)_eff_ (c), Im (χ_*sps*_^(2)^)_eff_ (d), and Im (χ_*ppp*_^(2)^)_eff_ (e) spectra at the air–water/formic acid mixture solution interface in the C–H stretch mode region with various *x*_*FA*_. (f) The amplitude *A*_*zzz*_^(2)^ as a function of the averaged depth of the chromophore. The solid lines and dotted lines are obtained using the approaches (i) and (ii), respectively, elaborated in the main text. The “×” marks denote the matching *A*_*zzz*_^(2)^ values inferred from the crossing points of the solid and dotted lines. (g) Comparison of the position shift of the C–H stretch chromophore between experiment and simulation.^30^ Copyright 2022 by the American Physical Society.

The *ε′* information can be connected with the averaged depth position of the vibrational chromophores.^16,40,67^ Let us consider the situation where the vibrational chromophores are located at *z* = *z'*. Here, a cavity containing the vibrational chromophores is embedded in the medium with the dielectric function of ε′ (see Figure 5a). The calculation of the local field correction^40^ leads to the expression of the interfacial dielectric constant *ε′* at position *z′*:^16^
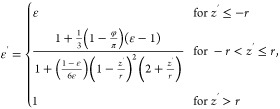
23where φ is the angle between the surface normal and the vector pointing from the center of the sphere to the crossing point of the dielectric interface and the sphere’s surface. *r* is the radius of the vibrational chromophore. *z* = 0 denotes the location where the chromophore experiences *ε′* = ε (ε + 5)/(4ε + 2) (φ = π/2).^16^ Note that φ = 0 and π/2 provide the interfacial dielectric constants within the Lorentz and Slab models, respectively.^16,68,69^Equation 23 links the interfacial dielectric function *ε’* with the depth position *z*. The variation of the dielectric constant described in eq 23 is displayed in Figure 5b, together with the MD simulation data.^40^ Despite the simplicity of the embedded model, it captures the trend that *ε′* varies with the depth position *z* on a ∼ 5 Å-scale. As such, one can get the Å-scale depth information from the PD-HD-SFG data.

As an example, we consider the depth location of formic acid molecules at the interface of air with a water/formic acid mixture, and vary the formic acid concentration (*x*_*FA*_). We chose formic acid as a benchmark molecule for demonstrating the validity of this scheme, because the C–H stretch mode can be easily assigned to the C–H group of formic acid, unlike the −CH_3_ group where the amplitude of the C–H mode is modulated by the Fermi resonance of the overtone of the H–C–H bending mode and C–H stretch mode.^70−72^ The measured Im (χ_*ssp*_^(2)^)_eff_, Im (χ_*sps*_^(2)^)_eff_, and Im (χ_*ppp*_^(2)^)_eff_ spectra are presented in parts c–e of Figure 5, respectively. The two approaches to reach the *A*_*zzz*_^(2)^ value outlined above are shown in Figure 5f. The matching of the *A*_*zzz*_^(2)^ value provides the average depth of the vibrational chromophores. The matching points of the *A*_*zzz*_^(2)^ values are marked by “×” in Figure 5f. This figure indicates that the average depth of the C–H stretch chromophore moves from the air region to the bulk region by ∼0.9 Å when the concentration of the formic acid changes from 2.5% to 10% molar fraction at the air–water/formic acid mixture. The trend that the C–H stretch vibrational chromophores of formic acid moves to the bulk with increasing *x*_FA_ is consistent with the AIMD simulation (Figure 5g). This result demonstrates that the PD-HD-SFG can capture the depth information with sub-Å-resolution.

Finally, we note that the probed region for the depth profile where the interfacial dielectric constant varies is |*z*| < ∼ 2 Å at the aqueous solution interface, while the SFG active region is at least |*z*| < 5 Å.^73−75^ As such, the probed region for the depth profile is thinner than the SFG active region. When wider probed region is required, using a novel technique to probe the nanometer scale depth profiling through the SFG and difference frequency generation spectra is available.^76^

## VI MD Simulation as a Tool for Critical Check of Experimental Result

Above, we outlined that several assumptions are required to interpret the SFG data. However, most of these assumptions cannot be accessed from the experimental side, meaning that computational support would greatly help.^52^ Computing the (multidimensional) orientational distribution from MD simulations is an essential guide for calculating the orientation of interfacial molecules, as seen above. Comparing the estimated depth from SFG measurement with the depth profile obtained from MD simulations is also very beneficial in guaranteeing the accuracy of the signal. Below, we explain the use of MD simulation for two cases.

**Scheme 1:**
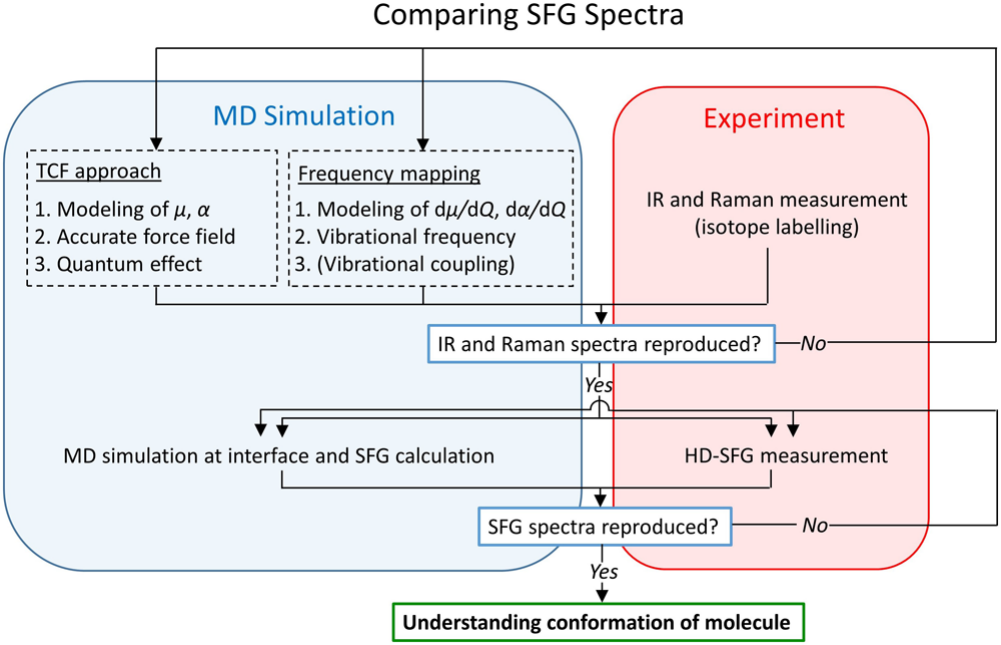
Typical Workflow for Comparing the SFG Spectra of Simulation and Experiment The model developed and testified with IR and Raman spectra^75,86−88^ are used for SFG spectra calculation, which is compared with the SFG experimental data.^49,51,53,89^

MD simulations have been used for computing spectra, allowing us not only to interpret vibrational spectroscopy data^52,77−80^ but also to check the accuracy of experimental data^53,81^ and modeling.^82,83^ Moreover, simulations can provide powerful support when making assumptions for the analysis of experimental data.^45,46^ The typical flow for computing the SFG spectra is displayed in Scheme 1. The IR and/or Raman spectra are first calculated, ensuring the accurate modeling of the vibrational frequency and (transition) dipole moment/(transition) polarizability by comparing with experimental data. Subsequently, the researchers tackle the SFG spectra simulation by using the frequency, dipole, and polarizability modeling developed for IR and Raman calculation. A typical drawback of this approach is that it is difficult to identify the origin of the discrepancy when the simulated SFG spectra differ from the experimental data. In this approach, the discrepancy of the spectra arises not only from the force field model used for MD simulation but also from the modeling of the dipole moment and polarizability used for computing spectra.

**Scheme 2:**
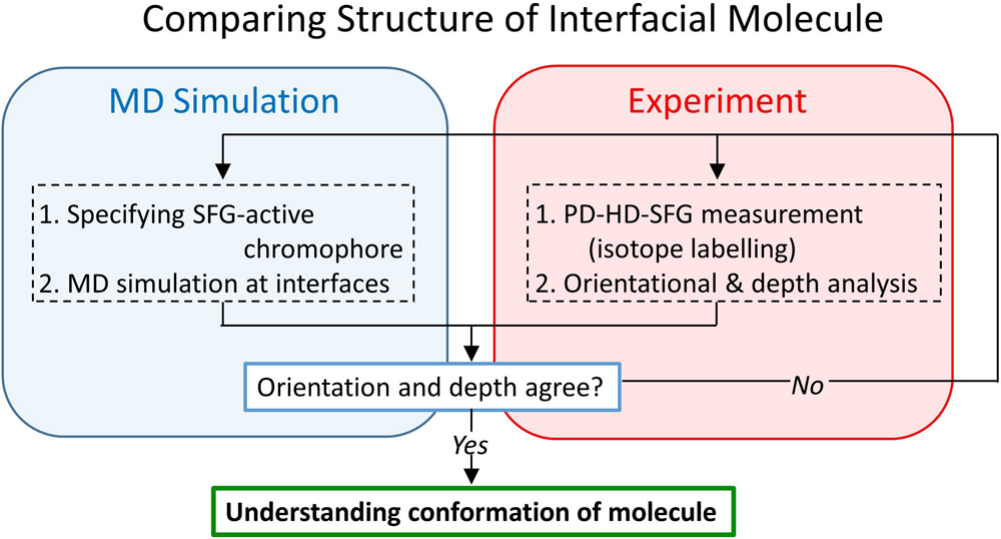
Workflow for Comparing the Structural Data of Interfacial Molecules Obtained from the PD-HD-SFG Spectra and the Simulation Data The interfacial structures of water,^45^ formic acid,^22^ and acetonitrile^46^ have been explored following this scheme.

PD-HD-SFG provides another route to compare the experimental SFG spectra with the MD simulation data.^4,26,84^ The flow of comparing the PD-HD-SFG data with the simulation data is described in Scheme 2. In this scheme, we can compare the experimental SFG data with the simulation data without performing the SFG spectra calculation, allowing us to skip computing the time evolution of the dipole moment and polarizability during the simulation. On the other hand, one should carefully pick the SFG-active species,^85^ for which one can calculate the orientational distribution and depth of the molecules from other criteria.

## VII Future Outlook

The PD-HD-SFG technique can be applied to explore the molecular-scale structure of liquid–liquid, liquid–solid, and air–solid interfaces. It opens the door to access the depth-related information in these interfaces. For example, it is interesting to understand how deeply the water molecules are in the oil subphase at the water–oil interface. Theoretically, it has been proposed that interfacial water forms a “finger-like” structure when ion transport occurs,^90,91^ but it has not been investigated experimentally, for lack of appropriate techniques. Furthermore, the technique can be used for identifying the SFG response of the hydroxyl group and their role in the wetting transparency.^80,92^

The depth profiling and orientational analysis through the PD-HD-SFG technique could be used for identifying the 3D structure of interfacial peptides or proteins, but this may require isotopic labeling of specific parts of the peptide or protein; all the amide modes in the peptide or protein backbones contribute to the SFG signal, making the individual position and orientation of the amide groups ambiguous. To resolve individual amide groups, isotope labeling of the target peptide and protein would be needed.^27,93,94^ Combining PD-HD-SFG with the isotope labeling is on the horizon.

## VIII Conclusion

In this Perspective, we explained how the PD-SFG technique can be used for understanding not only the molecular orientation but also the Å-scale depth profiling of molecules. Moreover, the technique can provide information on the interfacial dielectric constant profile. For these analyses, HD-SFG spectra with accurate phase determination are essential. Although the HD-SFG technique was first developed over 10 years ago, this technique has been rarely measured at the polarization combination other than *ssp* and has seldom been used for the analysis of interfacial molecular orientation. The HD-SFG measurement on the *sps*, *pss*, and *ppp* polarization combination and chiral polarization^95,96^ is on the horizon. Furthermore, such a PD-SFG technique has not been combined with the time-resolved SFG technique,^34,97−99^ except for some studies.^59,100−102^ Founding a theoretical basis for time-resolved PD-HD-SFG and its demonstration will be an interesting next challenge for the SFG community.
